# Specific Ion Effects in Hydrogels

**DOI:** 10.3390/molecules29245990

**Published:** 2024-12-19

**Authors:** Vincent Ball

**Affiliations:** Biomaterials and Bioengineering, Centre de Recherche en Biomédecine de Strasourg, Inserm UMR_S 1121, CNRS EMR 7003, Université de Strasbourg, 1 Rue Eugène Boeckel, F-67000 Strasbourg, France; vball@unistra.fr

**Keywords:** specific salt effects, kosmotropes, chaotropes, hydrogels, specific binding

## Abstract

Specific ion effects on the structure and function of many biological macromolecules, their associations, colloidal systems, interfacial phenomena, and even “simple” electrolytes solutions are ubiquitous. The molecular origin of such phenomena is discussed either as a salt-induced change of the water structure (the hydrogen bond network) or some specific (solvent mediated) interactions of one or both of the ions of the electrolyte with the investigated co-solute (macromolecules or colloidal particles). The case of hydrogels is of high interest but is only marginally explored with respect to other physico-chemical systems because they are formed through the interactions of gelling agents in the presence of water and the added electrolyte. In addition, hydrogels in a physiological environment, in which they are used for biomedical applications, may be subjected to fluctuations in their ionic environment. In this review, specific ion effects on the properties of hydrogels (made from macromolecules or small-molecular-weight gelators) are reviewed and discussed. In particular, the importance of specific ion binding to the molecules constituting the gel network versus the effect of the same ions on the structure of water is discussed.

## 1. Introduction

Even before the influence of electrostatic and Van der Waals non-specific forces on the stability of colloids was rationalized and quantified, the specific influence of salts, in particular anions, on the ability of proteins to precipitate or to crystallize from aqueous solutions was described [1]. Not only are the properties of biological macromolecules affected by the nature of the used electrolyte but also more simple interfaces. For instance, the interfacial tension of the water–air interface has been known for a long time to be salt-concentration-dependent. This property reflects negative adsorption (i.e., depletion) of some of the dissolved species at that interface according to the Gibbs adsorption isotherm. The molal surface tension increment is, however, strongly salt-dependent and follows the ranking predicted by the Hofmeister series (defined in Section 2); for instance, Na_2_SO_4_ displays a molal surface tension increment of 2.77 ± 0.09 $mN.m^{-1}.kg.{mol}^{-1}$ whereas NaClO_4_ at the other extremity of the Hofmeister series displays a value of 0.22 ± 0.06 $mN.m^{-1}.kg.{mol}^{-1}$ [2]. Similarly, the surface potential of aqueous solutions displays electrolyte-specific effects [3]. Surprisingly, some everyday life aspects like the degradation of wall paintings are influenced by specific ion effects [4].

Even if a great amount of empirical and theoretical knowledge has been acquired in the field of specific ion effects on biological processes and in colloid-interface science, the influence of the cations/anions in the Hofmeister series on the structure, gelation kinetics, and properties of hydrogels has been overlooked. Hydrogels are particularly interesting systems from this point of view because part of the water is used for the hydration of the gelling agents (macromolecules, particles, or small-molecular-weight gelators), and part is included in the porous volume of the hydrogel. There may be specific ion effects on both kinds of water molecules as well as on the gelling agents by themselves. It is the aim of this review to summarize the knowledge acquired in this field and to propose some experimental/theoretical perspectives to obtain deeper insights into salt-specific effects on hydrogel properties. Indeed, changing the properties of hydrogels by just playing on the ionic composition of water without adding some crosslinking reactions and avoiding the eventual toxicity due to the crosslinkers (glutaraldehyde, genipin, etc.) [5] would be of great benefit. In addition, hydrogels in biological environments may be submitted to changes in the ionic environment that may have beneficial or detrimental effects on their properties but are still largely unknown. This review is organized in the following manner: after having summarized the concepts used in ion specific effects, the available literature in the field on ion specific effects on hydrogels is described before some experimental and theoretical perspectives in this complex research field are proposed. The main research topic in this field is to know if the observed ion-specific effects are due to changes in the structure of water or (more likely) to specific interactions with the macromolecules used to form hydrogels.

## 2. Some Definitions to Describe Ion-Specific Effects

Even if many excellent reviews [6,7,8] are available to describe ion-specific effects on biological and on physicochemical phenomena, it is worthwhile to recall some definitions to help describe and understand them.

### 2.1. Kosmotropic Versus Chaotropic Ions

To describe salt-specific effects, ions have been classified as kosmotropes and chaotropes according to their water structure-making or structure-breaking influence [9,10]. On one hand, this classification now appears somewhat misleading [11] because it suggests implicitly that ions change the structure of the solvent (essentially the hydrogen bond network in water) on a macroscopic scale, i.e., beyond the first hydration shell of solvated ions. This change in the solvent structure induces in turn a modification of the macromolecular solvation or conformation. On the other hand, the ranking of the salts on the properties of a system (water and other solutes) is well established from a semi quantitative point of view. In a more recent view, kosmotropes are described as salting out compounds and chaotropes as salting in compounds, but the correlation between these two kinds of classifications is not always systematic [11].

It is also of note that a strong influence of the radial charge density of the anions but not of the cations was recently correlated with observed salt-specific effects [12].

**Scheme 1:**
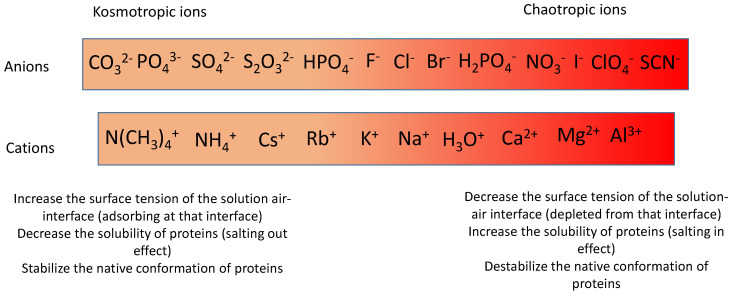
The main anions and cations and their classification in the Hofmeister series as well as some characteristic attributes of kosmotropic and chaotropic ions. Adapted from [14] with authorization.

Van Hippel and Wong established the influence of salt concentration on the melting temperature of ribonuclease by means of circular dichroism [13]. They established that KSCN and CaCl_2_ can be as efficient as neutral denaturants like urea and that the denaturing influence of guanidinium cations can be more than compensated by the stabilizing effect of sulfate anions. They also compared their data with those from other investigators who studied the melting temperature of DNA and myosin as a function of the supporting electrolyte [13]. The ranking of the cations and anions appeared very similar for all the investigated melting events. Such empirical findings allowed to establish the ranking of cations and anions shown in Scheme 1. This ranking is called the Hofmeister series.

### 2.2. Ionic Properties of Interest in Describing Ion-Specific Effects

Indeed, the degree of structure in water, a molecular concept, has consequences at a macroscopic level. From an empirical point of view, the dynamic viscosity *η* of an electrolyte solution is affected by the salt concentration *C* according to the following:

(1) ηη0=1+A.C12+B.C

In which $\eta_{0}$ is the dynamic viscosity of pure water at the same temperature at which the viscosity of the solution is measured. *A* and *B* are two constants [15]. The value of *A* reflects electrostatic nonspecific interactions, which can be described in the framework of the Debye–Hückel theory. *B* is the Jones–Dole viscosity coefficient and is aimed to quantify the amount of water that is structured. Positive *B* values correspond to an increase in viscosity when the electrolyte concentration increases, hence a water-structuring effect. Accordingly, *B* is positive for kosmotropic ions. Inversely, *B* is negative for electrolytes decreasing the solutions’ viscosity and decreasing the cohesion of water. This is the case of chaotropes.

The difficulty is that the measured viscosity of an electrolyte solution results from the contribution of the cations and of the anions. The chaotropic behavior of one ion can eventually be compensated by the kosmotropic behavior of its counterion. More details about the significancy of *B* coefficients and their values can be found in the review by Jenkins and Marcus [16].

**Figure 1:**
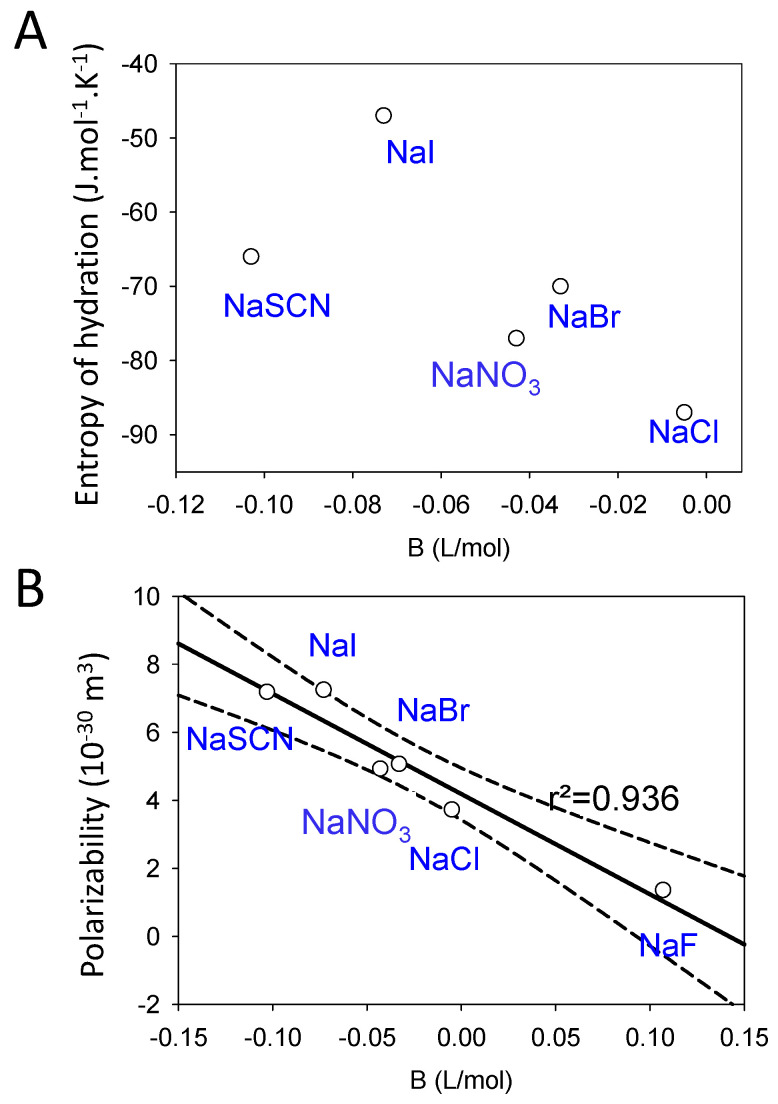
Correlation between the entropy of hydration and the viscosity *B* coefficient (**A**) and between the polarizability of the ion pair solubilized in water with the viscosity *B* coefficient (**B**). In panel B, the full and dashed lines correspond to a linear correlation and to the limits of the 95% confidence interval, respectively. The values of the viscosity *B* coefficients and of the entropy of hydration are taken from Table 2 of [18], whereas the polarizability values are taken from Table 1 of [19].

The influence of electrolytes on the structure of water can also be estimated by the entropy of solvation. In this approach, the contribution of both the cation and the anion are assumed to be additive. And for both the cation and the anion, the entropy change is due to the ion itself and the water. Only the second term (reflecting the water ordering) is of interest here. Kosmotropes, as estimated from the positive sign of the *B* viscosity coefficient, have a negative hydration component in their solvation entropy. The reverse holds for chaotropes [17]. On this basis, one would expect a good correlation between the values of the *B* coefficients and the entropy of hydration. This correlation exists from a qualitative point of view but is far from being excellent (Figure 1A). The correlation between the polarizability of the ion pair and the viscosity *B* coefficient is much better (Figure 1B). The polarizability can be used as a proxy for the ionic radius: the larger an ion is, the more it is polarizable.

Sodium perchlorate and sodium sulfate (where the anions are located at both extremities of the Hofmeister series, Scheme 1) at concentrations up to 6 $mol.L^{-1}$ have been shown not to have a significant effect on the hydrogen bond structure of water [20]. These data, obtained through femtosecond pump-probe spectroscopy, show that if ions have a structure-making or structure-breaking role in water, this effect can only concern the first solvation shell but not the whole bulk.

These data force the question of whether the ion-specific effects are related to specific anion (or cation) binding (adsorption) to the macromolecular solute whose properties are ultimately modified. Indeed, specific anion binding on macromolecules has been demonstrated for a long time on polyacrylamide beads using chromatographic experiments [21]. Interestingly, anion binding to poly(vinylpyrrolidone) has been investigated through ^127^I NMR line broadening. The competition between I^−^, Br^−^, and F^−^ to bind to the polymer allow the determination of the intrinsic binding constants of the anions to the polymer at 298 K (Table 1) [21].

As can be seen from Table 1, the anion binding constant of the anions to the selected polymer follows the expectations from the Hofmeister series: F^−^ < Cl^−^ < NO_3_^−^ ≈ Br^−^ < I^−^ < SCN^−^.

More recent evidence based on NMR spectroscopy also suggests specific ion binding to macromolecules in hydrogels [23,24,25].

The binding of anions to chiral amino acids was also used to explain their influence on the specific optical rotation of alanine, aspartic acid, glutamic acid, glutamine, proline, threonine, and tryptophan [26].

Since different properties of ions are correlated (as exemplified in Figure 1), it is of interest to correlate the macroscopic salt-dependent properties (melting temperature; kinetic parameters) to molecular attributes of the ions present in the electrolyte solution. Such kinds of correlations were made to rationalize the specific salt effects on hydrogel properties, as is shown in the following paragraphs.

## 3. Specific Ion Effects in Hydrogels Made from Polymers Displaying an LCST Behavior

Thermosensitive polymers have inspired increasing interest in developing stimuli-responsive materials and self-healing devices. For a long time and even now, poly(N-isopropylacrylamide) was considered as a model thermosensitive polymer owing to its temperature-sensitive hydration, collapsing above a critical solution temperature for entropic reasons but behaving as a hydrogel below that temperature. The lower critical solution temperature (LCST) of poly(N-isopropylacrylamide) (PNIPAM) decreases from 32–33 °C in pure water to about 10 °C when the NaCl concentration is increased to 2 $mol.L^{-1}$. The effect of sulfate (divalent) anions is much more pronounced because an LCST at 10 °C is obtained already at about 0.45 $mol.L^{-1}$ in sodium sulfate [27].

**Figure 2:**
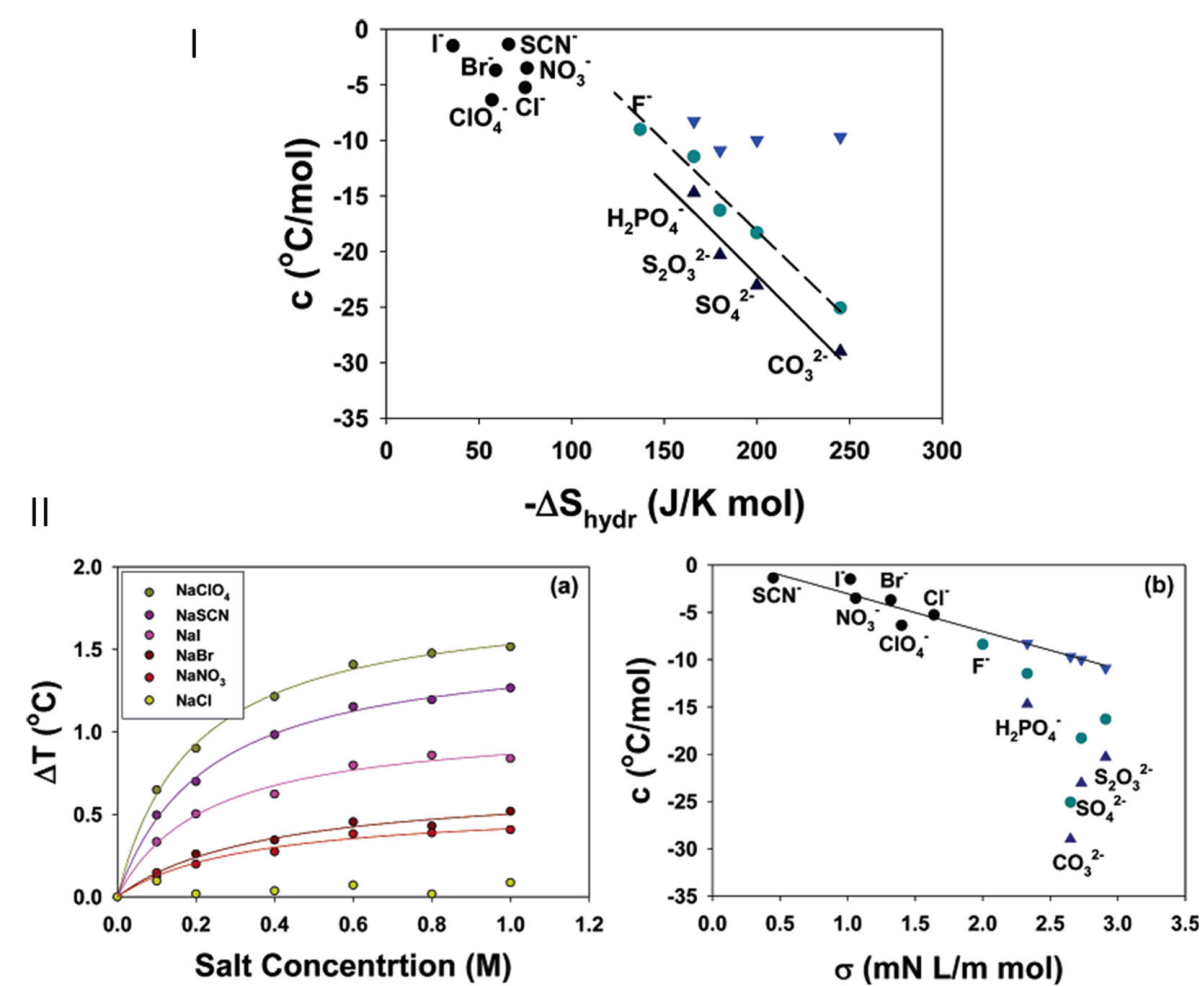
(**I**) Depression of the LCST of PNIPAM versus the hydration entropy of the used anion. A good correlation is found for kosmotropes (full and dashed lines, the nature of the anions in the form of a sodium salt) but not for chaotropes, which are clustered in the upper-left part of the figure. (**IIa**) Change in the LCST of PNIPAM for sodium salts of chaotropic anions. The lines correspond to the fits of Langmuir isotherms to the experimental data. (**IIb**) Depression of the LCST of PNIPAM versus the surface tension increment of the aqueous electrolyte solutions. This time, the correlation is good for chaotropes (full line, the nature of the anions in the form of a sodium salt) and bad for kosmotropes, which are clustered in the lower-right part of the figure. Reproduced from [28] with authorization.

In a more recent study [28], the effect of anions in sodium salts on the LCST of PNIPAM was rationalized as the sum of two contributions, the first one being related to the hydration entropy of the used anions and the second one being related to specific anion binding on the polymer. The first contribution was found predominant in the case of kosmotropes and the second in the case of chaotropes (Figure 2).

The hydrodynamic diameter and the electrophoretic mobility of both negatively and positively charged PNIPAM microgel particles have been measured as a function of the temperature and the ionic strength in the presence of NaCl, NaNO_3_, NaSCN, Ca(NO_3_)_2_, and NH_4_OH [29]. It appeared that the electrophoretic mobility measured as a function of temperature was highly salt-dependent even at 0.01 M ionic strength for both the anionic and cationic microgels above the lower critical solution temperature. In the case of the cationic microgel, the electrophoretic mobility became negative in the presence of NaSCN, indicating the occurrence of surface charge reversal in relation to strong adsorption of thiocyanate anions. Overall, the effect of the anions (Cl^−^, NO_3_^−^, and SCN^−^ in the form of a sodium salt) was much more pronounced than that of the cations (Na^+^, NH_4_^+^, Ca^2+^, and NO_3_^−^ being the common anion). This finding is pretty general in specific ion effects and is attributed to the higher polarizability of the anions compared to the cations [30]. Below the LCST, when the particles were in a swollen state, almost no specific ion effect was found concerning the particles’ electrophoretic mobility. However, below the LCST, the particles’ hydrodynamic diameter was salt-dependent at ionic strengths above about 200 mM, when the electrostatic interactions are largely screened. The particles collapsed at this high ionic strength according to the following sequence: NaCl > NaNO_3_ > NaSCN. This effect may be due to a change in the solvent structure immediately around the microgel particles induced by the salt. Hence, this interesting work shows the combination of two salt-specific mechanisms: a specific adsorption and an influence on the local water structure. In a similar trend but relying on small-angle neutron scattering, the counterion cloud around PNIPAM microgels was found to be different between NH_4_^+^ and Na^+^ as counterions. This finding strongly suggests that the ion specificity originates from some binding or ion condensation in this particular system [31]. Using the same technique, SCN^−^ anions were shown to bind to polyethyleneoxide but not to ethylene oxide monomers and their small oligomers, implying the importance of sufficiently long chains to allow for specific anion binding effects [32].

The gelation of cellulose nanofibril/PNIPAM gels was investigated in the presence of sodium and calcium chloride [33], with an important cation-specific influence in screening the electrostatic repulsions between the cellulose nanofibrils, hence increasing the gels’ stiffness.

To summarize, marked specific ion effects on the swelling properties of hydrogels made from thermosensitive polymers have been observed and strongly correlated to ion adsorption on the polymer chains.

## 4. Specific Anion Effects on Other Hydrogels

### 4.1. Polymer-Based Hydrogels

#### 4.1.1. Protein-Based Hydrogels

Protein-based hydrogels, mainly made from the denatured form of collagens, namely gelatins, are important materials in the food industry and for medical care [5]. They often need to be crosslinked to improve their mechanical properties and long-term stability. But such crosslinking chemistry most of the time has issues from the point of view of cytotoxicity. In this context, a deep investigation of the use of electrolytes to tailor gelatin-based hydrogels’ properties has become of the highest interest.

The influence of electrolytes on the melting point (the inflection point on the gel-to-sol transition curve) of gelatin was described early in the literature. The influence of specific ion binding was assumed to play a major role based on experiments made with chemically modified gelatin [34]. The triple-helix content of gelatin (Type B, Bloom number of about 220 g) gels in the presence of 100 mM potassium salts decreased in the following sequence: K_2_SO_4_ > KH_2_PO_4_ > KCl >> KSCN, in relationship with a decrease in the gel’s storage modulus G′ [35]. Those finding are in perfect agreement with the predictions based on the Hofmeister series (Scheme 1). It appears that the chaotropes hinder the triple-helix formation, which is at the origin of the gelation process [36].

It is well known that gelatins from different origins have different amino acid compositions and different molecular weights, effecting their gelation ability but apparently not the occurrence of specific salt effects. Indeed, tilapia fish gelatin gels (10% *w*/*v*) were immersed in (NH_4_)_2_SO_4_ solutions at increasing concentrations, which led to an increase in the hardness of the hydrogels as well as an increase in their storage modulus. Even if the effect of other electrolytes was not investigated, this study highlighted that the combination of a strong kosmotropic cation and a strong kosmotropic anion leads to a strong reduction in the water mobility inside the gels (T_2_ relaxation times measured by NMR) and to an important morphological change (SEM) with a decreasing pore size upon a salt concentration increase. In addition, an increase in the hydrogen bonds formed by the water molecules (with respect to pure water) was found by FTIR spectroscopy [37].

**Figure 3:**
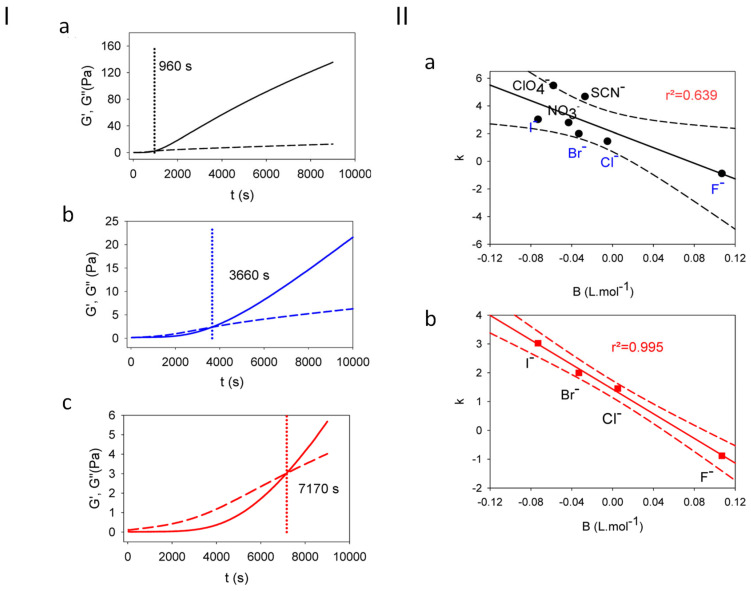
(**I**) Typical gelation kinetics of 10% (*w*/*v*) bovine gelatin solutions in the presence of sodium salts at an ionic strength of 100 mM: (**a**): NaCl, (**b**) NaI, and (**c**) NaSCN. (**IIa**) variation of the constant *k* (defined in Equation (2) as the proportionality constant between the logarithm of the ionic strength and the logarithm of the gelation time) as a function of the Jones and Dole viscosity *B* coefficient of the different used sodium salts (indicated on each experimental point) in the gelatin solutions. (**IIb**) The same data as in panel (**IIa**) but restricted to halide anions. The full and dashed lines correspond to linear regressions and to the limits of the 95% confidence interval, respectively. Modified from [19] with authorization from the American Chemical Society.

From a dynamic point of view, the gelation kinetics of bovine gelatin (10% *w*/*v*) were investigated as a function of the salt concentration for different sodium salts of monovalent anions. It was found that sodium fluoride allowed to decrease the gelation time (measured by shear rheometry at 25 °C) in a concentration-dependent manner. All the other sodium salts slowed down the gelation kinetics in a similar concentration-dependent manner. The effect was more pronounced in the following sequence: Cl^−^ < Br^−^~NO_3_^−^ < SCN^−^~ClO_4_^−^ [19] (Figure 3I), meaning that the most chaotropic anions slow down and eventually inhibit the gelation process. From a more quantitative point of view, the gelation time τ (defined at the duration when the storage modulus G′ becomes equal to the loss modulus G″, characterizing viscous losses in the hydrogel) could be fitted as a function of the ionic strength of the solution according to the following:

(2) $$log\tau =log\tau_{0}+k.logI$$

where *k* represents the specific effect of a given anion. The *k* values increased markedly with the chaotropic character of the used anion and were strongly correlated with the polarizability of the used anion and its viscosity *B* coefficient (Figure 3II). Note that G′ characterizes the elastic response of the hydrogel, namely the contribution of the shear stress, which is in phase with the applied strain, whereas the contribution of the shear stress in quadrature with the applied stress allows the calculation of G″.

Cations (chlorides) have also a specific effect on the gel strength (the storage modulus measured 500 s after having cooled the hot sol to 25 °C), the melting point, and the gelation time of porcine gelatin hydrogels [38]. The effect of divalent cations was more pronounced than that of monovalent cations. The gel strength, the melting point, and the gelation time were all found to scale with the ionic strength according to a power law with salt-specific decay constants. These decay constants were found to scale linearly with the ionic radius of the cations [38], which are directly related to their polarizability (at least for spherical ions). The gels investigated in references [19,38] were prepared by allowing a hot gelatin solution, in the presence of the selected electrolyte, to gel. The specific ion effects seemed, however, not to appear during the gelation process but occurred in solution, as was nicely demonstrated. Indeed, diluted gelatin solutions in the presence of potassium salts of citrate, SO_4_^2−^, H_2_PO_4_^−^, CH_3_COO^−^, Cl^−^, and SCN^−^ were investigated by means of viscosimetry, optical rotation, and dynamic light scattering. The intrinsic viscosity of the gelatin solutions was found to increase according to the chaotropic nature of the anions in the Hofmeister series. Simultaneously, the specific rotation of gelatin decreased in the same sequence, revealing the tendency of chaotropic anions to decrease the triple-helix content of gelatin, explaining the lower tendency of gelatin to gel or to form weaker gels in the presence of chaotropes [39]. Interestingly, the salt specificity of gelatin hydrogels remained even after chemical crosslinking [40].

Finally, concerning gelatin mixed with sodium alginate, the influence of anions on its self-healing properties was investigated. The swelling degree of those mixed gels was found to be higher in the presence of a given salt concentration according to the following sequence: NaCl > NaNO_3_ > Na_2_SO_4_ > NaCH_3_COO. As expected, the reverse sequence was found for the storage moduli obtained from frequency sweep experiments. The self-healing properties of the used hydrogels were also investigated and were found to be improved in the presence of the chaotropic NO_3_^−^ and Cl^−^ anions with respect to hydrogels containing kosmotropic CH_3_COO^−^ and SO_4_^2−^ anions [41].

Bowland and Foegeding investigated the rheological, morphological, and optical (turbidity) properties of whey protein isolate hydrogels as a function of the ionic strength of the aqueous solution in the presence of Na_2_SO_4_, NaHPO_4_, and NaSCN. Their main result was that the transition from a fine-stranded morphology to a particulate morphology, occurring at higher ionic strengths, was retarded in the presence of NaSCN, the salt containing the most chaotropic anion. Simultaneously, the gels made in the presence of NaSCN were less turbid, reflecting the presence of fewer or smaller aggregates than their Na_2_SO_4_ or NaHPO_4_ counterparts [42].

Recently, protein isolate hydrogels were produced by thermal denaturation yielding to either fine-stranded or particulate-based hydrogels. The obtained gels were then incubated in sodium salt solutions with different anions from the Hofmeister series. Kosmotropic anions like sulfate induced a significant increase in the Young’s modulus, whereas chaotropic anions like iodide induced significantly lower moduli in the case of hydrogels with a fine-stranded microstructure, but the influence of the anions was minor in the case of hydrogels with a particulate microstructure [43].

To summarize, ion-specific effects on protein-based hydrogels (mainly gelatin- and whey protein isolate-based materials) were evidenced but far fewer investigations compared to PNIPAM hydrogels have been aimed at characterizing the molecular origin of those effects.

#### 4.1.2. Polysaccharide-Based Hydrogels

Polysaccharides are the most abundant biopolymers on earth, with a plethora of biological functions, and they are also directly used or chemically modified for real-life applications, particularly in the food industry. Some of them, like agarose, pullulans, and carrageenans, are able to gel water.

Salt effects of the gelation of agarose (induced by a temperature decrease from aqueous agarose solutions) have been investigated by means of turbidity and circular dichroism measurements [44]. It was found that NaSCN delays the onset temperature of gelation in a salt concentration-dependent manner, and in all cases, the gelation occurs at much lower temperature than in the presence of the same concentration of NaCl. The helicity of agarose is also negatively affected by the most chaotropic anions, as in the case for gelatin hydrogels [45]. The data concerning agarose have been interpreted as binding of thiocyanate to the agarose chains in the framework of the Schellman model [46]. The fit of the theoretical model to the experimentally determined temperature of gelation onset was excellent. The assumption of preferential SCN^−^ binding to agarose with respect to Cl^−^ binding was confirmed by means of NMR relaxation experiments [47].

Surprisingly, for a gel made from uncharged macromolecules, the mechanical properties like the shear and Young moduli of agarose hydrogels (1% *w*/*v*) decreased with an increase in the ionic strength, the used electrolytes being LiCl and NaCl. Above about 0.5 M in ionic strength, the shear and loss moduli were significantly lower in the presence of the salt containing the lost chaotropic cation, namely LiCl. In addition, when the gels were compressed up to 15% and relaxed, those formed in the presence of NaCl dissipated more energy. Interestingly, the more rigid NaCl-incubated gels allowed for a lower incorporation of bovine serum albumin. Of the highest interest was the finding that once the agarose gels were set in the presence of either LiCl or NaCl, their mechanical properties did not return to those found in pure water when the gel is intensively rinsed with the pure solvent [48].

Contrarily to the uncharged agarose gels for which the melting temperature is anion-specific with almost no influence of the cation, the melting temperature of the sulfated and hence negatively charged kappa-carrageenans is cation-dependent, with almost no influence of the used anion [49]. The melting temperature, measured as the inflection point in the specific rotation versus the temperature curve, varied according the following sequence: Rb^+^ > Cs^+^ > K^+^ > NH_4_^+^ > N(CH_3_)_4_^+^ >Na^+^ > Li^+^ [49], almost in agreement with a reverse Hofmeister effect. Note that a reverse Hofmeister effect corresponds to an increased solubility or a more swollen state in the presence of kosmotropes. In the experimentally determined sequence, the sodium and the lithium cation are ranked at a position that does not correspond to the Hofmeister series (Scheme 1). The strong influence of Rb^+^ was interpreted with the Manning counterion condensation theory [50]. The agreement with Manning’s theory implies specific binding depending on the charge density of the cation and the average distance between charged groups on the polyelectrolyte (here, kappa-carrageenan). For the same macromolecules, the storage modulus of 1.5% (*w*/*w*) hydrogels was also found to be anion-independent but depending on the cation used in the electrolyte solution, with the storage modulus decreasing in the following order: Cs^+^ > K^+^ >> Na^+^ ≈ Li^+^ (Cl^−^ being the common anion) [51], hence following the lyotropic series (Scheme 1). Those data were interpreted in terms of the influence of the cations on the water structure, hence a totally different interpretation than that proposed by Rochas and Rinaudo [49].

**Figure 4:**
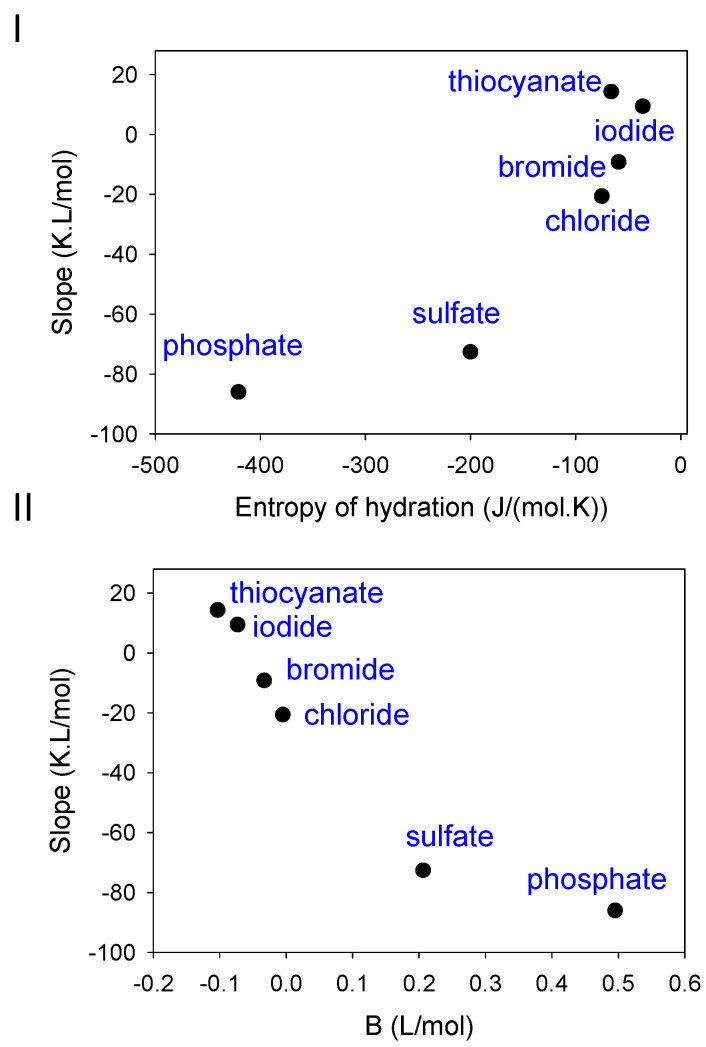
Correlation between the slope of the peak temperature measured during DSC scans on hydroxypropylmethylcellulose hydrogels as a function of the salt concentration versus the entropy of hydration (**panel I**) and the viscosity *B* coefficient (**panel II**). For all those anions (indicated in the figures), sodium was the common cation. Data taken from Table II of [45].

The gelation of hydroxypropylmethylcellulose in the presence of different sodium salts has also been investigated by means of differential scanning calorimetry and rheology [45]. Interestingly, the peak in the endothermic gel–sol transition, *T_p_*, varied linearly with the salt concentration in a salt-specific manner. The slope of *T_p_* versus the salt concentration curve correlates with the Jones–Dole *B* viscosity coefficient and the entropy of hydration but not linearly (Figure 4). The correlation would be close to linear if phosphate anions were not taken into account. Those extremely kosmotropic anions, referring to the highly negative hydration entropy and the highly positive viscosity *B* coefficient, do not depress the slope of the melting temperature versus salt concentration as much as expected when relying on the trend displayed by the other anions. Even if the authors of the original paper did not provide an explanation for the correlation displayed in Figure 4, it is assumed herein that the chaotropes may bind to hydroxypropylmethylcellulose and change its heat capacity, hence the effect measured by differential scanning calorimetry.

From the point of view of the occurrence of binding between the cations in the Hofmeister series and κ-carrageenan, ^39^K NMR spectroscopy on κ-carrageenan gels was performed. The data show that K^+^ is bound to the polymer in the gel state and that the binding is significantly lowered above the gel–sol transition temperature. When KCl is replaced by KI, containing a more chaotropic anion, the binding of the K^+^ is markedly decreased [23].

In more complicated systems, mixed gels made from soy protein isolate and κ-carrageenan were immersed in K_2_SO_4_, KCl, and KSC solutions at different concentrations for 12 h (at 4 °C). Their rheological properties were subsequently measured between 0.1 and 18 Hz at a fixed strain of 0.1%. In the whole investigated frequency range, the storage modulus varied according the following sequence: SO_4_^2−^ > Cl^−^ > SCN^−^, hence in agreement with the predictions from the Hofmeister series [52].

Finally, concerning alginate hydrogels, the mechanical properties are dominated by the nature of the used cation, which indeed induces gelation. The Cu^2+^-based hydrogels have a higher storage modulus (around 250–300 kPa depending on the copper salt concentration) than the Zn^2+^- and Ca^2+^-based hydrogels (between 20 and 100 kPa). In addition, the nature of the counter anion (CH_3_COO^−^, Cl^−^, or SO_4_^2−^) plays a significant role. Furthermore, for each metallic cation in the salt, the storage modulus of the alginate hydrogels increased in the following order: CH_3_COO^−^ > Cl^−^ > SO_4_^2−^. The authors recognized by themselves that the investigation needs to be extended to other anions in the Hofmeister series [53].

It appears that many hydrogels made from polysaccharides display ion-specific properties. For such materials, many investigations [46,47,49] point to the importance of specific cation or anion adsorption to the biopolymer. However, some articles led to contradictory conclusions [49,51], illustrating the need for complementary investigations with such gels.

#### 4.1.3. Block Copolymers-, Polyampholyte-, and Synthetic Polymer-Based Hydrogels

**Figure 5:**
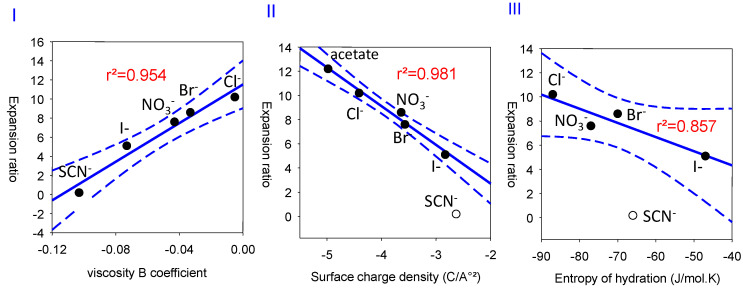
Correlations between the expansion ratio of poly(methyl methacrylate)_88_-block-poly(2-diethylamino)ethyl methacrylate)_223_-poly(methyl methacrylate)_88_ as measured by SAXS. (**I**) Influence of the viscosity *B* coefficient, (**II**) influence of the surface charge density, and (**III**) influence of the entropy of hydration. The full lines correspond to linear regressions and the dashed lines to the 95% confidence intervals. The data for the thiocyanate anion (open circles in part II and III) have not been taken into account for linear regression. Reproduced from [18] with authorization.

Finally, block copolymers can also be designed to allow for water gelation. As a recent example, the pH-induced swelling of poly(methyl methacrylate)_88_-block-poly(2-diethylamino)ethyl methacrylate)_223_-poly(methyl methacrylate)_88_ in the presence of sodium salts was investigated by means of small-angle X-ray scattering (SAXS) [18]. Upon swelling, the diffraction peak shifted to smaller wavenumbers, which corresponds to a size increase of the swollen central domain of the triblock copolymer. In turn, this allowed the calculation of the expansion ratio of the gel domains, which followed a *reverse Hofmeister effect*: a strong expansion in the presence of 0.1 M sodium acetate and almost no expansion in the presence of the chaotropic sodium thiocyanate. The expansion ratios were plotted versus the surface charge density of the anions, the viscosity *B* coefficient, and the entropy of hydration (Figure 5).

Interestingly, the correlation between the expansion ratio and the viscosity *B* coefficient was excellent for all the investigated salts, but sodium thiocyanate fell out of the correlation concerning the surface charge density and the entropy of hydration [18].

Quartz crystal microbalance with dissipation monitoring was used to investigate the swelling and rheological properties of another copolymer-based hydrogel. This hydrogel was made from 90% *N*,*N*-dimethylacrylamide and 10% 2-(N-ethylperfluorooctane sulfonamido) ethyl acrylate (FOSA), which leads to hydrophobic domains in the hydrogel. This material was deposited by spin coating on a quartz crystal and subsequently annealed at 150 °C for 18 h to obtain a 100 nm thick film. The hydrogel was then allowed to swell in water and was subsequently put into Na_2_SO_4_, NaBr, and NaClO_4_ solutions of increasing concentrations. In the presence of the kosmotrope Na_2_SO_4_, the swelling of the gel decreased upon an increase in the salt concentration, and the complex shear modulus increased monotonously, as expected for less swollen gel. The intermediate salt in the Hofmeister series, NaBr, induced only a small reduction in swelling and almost no changes in the rheological properties upon salt concentration increase when compared to pure water. In total contrast, in the presence of the most chaotropic salt, NaClO_4_, the film swelling increased up to about 0.02 $mol.L^{-1}$ in salt concentration, as expected for a salting-in effect, but it decreased for higher salt concentrations. The changes in the complex shear modulus only weakly correlated with the change in the swelling behavior in the presence of NaClO_4_ [54].

To provide possible applications in energy storage devices, polyampholyte hydrogels made from the copolymerization of sodium-4-vinyl-benzenesulfonate (as the anionic monomer) and [3-(methacryloylamino)-propyl] trimethylammonium chloride (as the cationic monomer) were intensively dialyzed to remove all the internal counterions before a second dialysis step in the presence of LiCl, NH_4_Cl, NaCl, KCl, MgCl_2_, and CaCl_2_, namely electrolytes with a common anion and variable cations. The same experiments were realized for sodium salts of variable anions: Na_2_SO_4_, Na_2_HP0_4_, NaCl, NaNO_3_, and NaClO_4_. It was found that the polyampholyte hydrogel swelling was strongly influenced by the nature of the anions and much less by changing the cation in the electrolyte solution (but with some small counterintuitive effects). The swelling effect of the anions followed the ranking expected on the basis of the Hofmeister series. Interestingly, the ionic conductivity also increased with the chaotropic nature of the used anion in the electrolyte. As expected, the ionic conductivity of the hydrogels, determined from electrochemical impedance spectroscopy, increased with the concentration of the used electrolytes but without a dramatic deterioration of the gels’ elasticity. From this point of view, those polyampholyte hydrogels are superior to the poly(vinyl alcohol)-based hydrogels traditionally used as gel polymer electrolytes in energy storage devices [55].

Concerning hydrogels made from synthetic polymers, the storage modulus of poly(methacrylamide) hydrogels can be changed by four orders of magnitude by changing the nature of the sodium salt or chloride salt in which it is immersed. The stiffest but also the more brittle hydrogels were obtained in the presence of sodium citrate, Na_2_SO_4_, as well as in the presence of N(CH_3_)_4_Cl. The rheology data could be analyzed on the basis of a time–salt superposition curve, and the small-angle X-ray scattering curves of the hydrogels showed a marked difference between the gels immersed in solutions containing chaotropic ions and in solutions containing kosmotropic ions. In the case of the kosmotropes, the scattered intensity scaled according to a q^−4^ law at low scattering vectors, suggesting the occurrence of phase separation inside the hydrogel. Interestingly, the obtained hydrogels showed a reversible character when immersed successively in Na_2_SO_4_ (turbid and rigid) and in NaSCN solutions (transparent and soft) [56].

Finally, as a recent example of salt-specific effects on inorganic–polyelectrolyte hybrid hydrogels, Laponite was gelified with the addition of polyitaconate, whose carboxylic groups were titrated with stoichiometry amounts of LiOH, NaOH, and KOH. The storage modulus of the obtained hydrogels varied in the following order: Na^+^ < Li^+^ < K^+^, hence not according to the ranking expected on the basis of the cation Hofmeister series: Li^+^ < Na^+^ <K^+^. This finding was rationalized using multinuclear NMR experiments. It appeared that all the K^+^ cations but only 68% of the Na^+^ were strongly bound to the polyitaconate chains, with almost no detectable bonding of Li^+^ to the polyanion. The peculiar behavior of Na^+^, producing weaker gels than the other counter cations, was then attributed to its higher diffusion coefficient as measured by diffusion-ordered NMR spectroscopy [25].

It appears that relatively few investigations have been devoted to the characterization of hydrogels made from block copolymers and polyampholytes. Additional studies are required in the future, particularly to characterize the occurrence (or not) of specific adsorption phenomena.

### 4.2. Small-Molecular-Weight Gelators

Hydrogels made from synthesized small-molecular-weight gelators may present some significant advantages with respect to their counterparts made from polymers, particularly a higher sensitivity to external stimuli.

A proline-modified calix[4] arene was shown to undergo gelation between pH 0 and 7 in an anion-specific manner but disassembled above pH 7. No gelation was observed in the presence of the kosmotropic sodium sulfate anion, whereas gelation occurred in the presence of sodium chloride, nitrate, bromide, iodide, and perchlorate, hence in the presence of chaotropic anions [57]. This trend is totally different from that found for gelatin [19,35,38] and agarose hydrogels [44,47]. However, the gels obtained in the presence of 0.1 M of the most pronounced chaotropes were only transiently stable and ended with the formation of crystals [57]. For a given anion inducing gelation, the hydrogel qualitative mechanical properties (ability to withstand its own weight) were modulated by the nature of the counteraction in the electrolyte.

The gelation of 1-(3-methyl-1H-pyrazol-5-yl)-3-(3-nitrophenyl)urea yielded to hydrogels in a narrow pH range between 1 and 2, with the storage modulus decreasing in the following order: H_2_SO_4_ > MePO_3_H_2_ > H_3_PO_4_ ≈ HBF_4_ ≈ HPF_6_ > EtPO_3_H_2_ [58]. This investigation is interesting due to the use of anions (BF_4_^−^; PF_6_^−^), which are only rarely investigated for their specific effects and do not even appear (systematically) in the Hofmeister series, as shown in Scheme 1.

Small-molecular-weight gelators made from L-valine dipeptides were shown to display anion- and cation-specific solubility and mechanical properties following the qualitative ranking predicted by the Hofmeister series [59]. In addition, when guanidinium hydrochloride was used as the supporting (chaotropic) electrolyte, ^13^C NMR spectroscopy allowed to demonstrate the occurrence of binding between the cation and the carbonyl group of the gelator [59]. The release of Alizarin yellow from the hydrogels was the result in the presence of different salts, and it was found to be faster and more pronounced in the presence of chaotropic cations or anions.

The morphology of N-alkyl-n-(R)-12-hydroxyoctadecyl ammonium salts with hydrogels containing Cl^−^, Br^−^, NO_3_^−^, and BF_4_^−^ was found to be anion-dependent [60].

## 5. Discussion and Conclusions

The different properties of hydrogels, like the conformational state of their constituent macromolecules, their melting temperatures, their gelation times, and their storage moduli, are most often salt-specific (at a given ionic strength in the aqueous solution). The observed salt-specific properties have been correlated to molecular properties of the variable anion or cation (at constant cation or anion, respectively) in some instances [18,19,45]. The correlations are often good but with some exceptions, as shown in Figure 5 in the case of thiocyanate anions in the presence of poly(methyl methacrylate)_88_-block-poly(2-diethylamino)ethyl methacrylate)_223_-poly(methyl methacrylate)_88_ microgels [18]. The correlation is often much better when the investigated anion in the Hofmeister series is spherical (compare panels IIa and IIb in Figure 3) [19]. In some instances, as for kappa-carrageenans, the measured cation influence on the melting temperature does not exactly follow the Hofmeister series [49], and there are even some small inconsistencies between different studies on the same gelator [49,51]. But, at the present stage, one cannot exclude that such inconsistencies originate more from the different molecular weight or the fraction of sulfated groups in kappa-carrageenans than from specific cation effects.

In some instances, the observed specific effect of the anions (for a constant cation) has been clearly attributed to some small preferential adsorption to the gelator [21,25,28,47,49]. Those findings increase the validity of the assumption that specific salt effects on hydrogel properties (as well as for other systems) are due to specific (but weak) preferential interactions between ions and the gelator, even in the case of small-molecular-weight gelators [59]. Even in the case where the occurrence of specific binding has not been explicitly demonstrated, the linear change of the measured ion-specific parameter with the polarizability of the ion [19] is a strong indication of the occurrence of “binding” because the intensity of dispersion forces scales with the polarizabilities of the interacting molecules [61].

Even if more investigations should be performed to highlight the influence of specific salt effects on the properties of hydrogels, it seems even more important to address the questions of the reversibility of salt effects. There have only been a few investigations, to the author’s knowledge, of hydrogels prepared in the presence of an electrolyte E_1_ and subsequently immersed in electrolyte E_2_ (of the same ionic strength as E_1_): will the properties (storage modulus, etc.) of the hydrogel be identical to those of the gel directly prepared in the presence of E_2_ and vice versa? If there is reversibility, what is the rate of change? Is the effect dependent on the ionic strength? Indeed, considering the low ion binding constant values already published (Table I, [22]), one may expect important exchange between ions of similar affinity for the gelling agent. However, the slow water motion between the different comportments of the gels may considerably slow down the diffusion of ions between them. Experimentally, some reversibility was found in the case of hydrogels synthesized with ammonium persulfate during the radical polymerization and subsequently exchanged with other electrolytes [31]. Similarly, poly(methacrylamide) hydrogels showed a reversible change when immersed successively in Na_2_SO_4_ (turbid and rigid) and in NaSCN solutions (transparent and soft) [56]. On the other hand, some examples of irreversible salt effects have been noticed: when agarose hydrogels are set in the presence of either LiCl or NaCl, their mechanical properties do not return to those found in pure water when the gel is intensively rinsed with the pure solvent [48].

Overall, more systematic investigations to directly quantify the ion adsorption on gelling agents should be undertaken because such investigations could be useful not only in the field of hydrogels but more generally for understanding the mechanism(s) behind Hofmeister effects. For instance, the influence of chaotropic anions on the depression of the LCST of PNIPAM has been interpreted with a model relying on the Langmuir adsorption isotherm, strongly suggesting the adsorption of those anions (with a weak affinity) on the amide groups of PNIPAM [28].

## Figures and Tables

**Table 1:** K between anions and poly(vinylpyrrolidone). Data taken from [22].

Anion Type	*K*
F^−^	0.1
Cl^−^	0.5
Br^−^	2.4
I^−^	3.3
NO_3_^−^	1.9
SCN^−^	5.3

## Data Availability

Dataset available on request from the author.
